# Modelling the potential of focal screening and treatment as elimination strategy for *Plasmodium falciparum* malaria in the Peruvian Amazon Region

**DOI:** 10.1186/s13071-015-0868-4

**Published:** 2015-05-07

**Authors:** Angel Rosas-Aguirre, Annette Erhart, Alejandro Llanos-Cuentas, Oralee Branch, Dirk Berkvens, Emmanuel Abatih, Philippe Lambert, Gianluca Frasso, Hugo Rodriguez, Dionicia Gamboa, Moisés Sihuincha, Anna Rosanas-Urgell, Umberto D’Alessandro, Niko Speybroeck

**Affiliations:** Instituto de Medicina Tropical Alexander von Humboldt, Universidad Peruana Cayetano Heredia, Lima 31, Peru; Department of Biomedical Sciences, Institute of Tropical Medicine, Antwerp, 2000 Belgium; Research Institute of Health and Society (IRSS), Université catholique de Louvain, Brussels, 1200 Belgium; Department of Medical Parasitology, New York University, New York, 10012 USA; Institut des sciences humaines et sociales, Université de Liège, Liege, 4000 Belgium; Región de Salud Loreto, Iquitos, Loreto 160 Peru; Departamento de Ciencias Celulares y Moleculares, Facultad de Ciencias y Filosofia, Universidad Peruana Cayetano Heredia, Lima 31, Peru; Facultad de Medicina, Universidad Nacional Amazonia Peruana, Iquitos, Loreto 160 Peru; Disease Control and Elimination, Medical Research Council Unit, Fajara, 220 The Gambia; London School of Hygiene & Tropical Medicine, London, WC1E 7HT UK

**Keywords:** Malaria, *Plasmodium falciparum*, Focal Screening and Treatment (FSAT), Modelling, Elimination, Peru

## Abstract

**Background:**

Focal screening and treatment (FSAT) of malaria infections has recently been introduced in Peru to overcome the inherent limitations of passive case detection (PCD) and further decrease the malaria burden. Here, we used a relatively straightforward mathematical model to assess the potential of FSAT as elimination strategy for *Plasmodium falciparum* malaria in the Peruvian Amazon Region.

**Methods:**

A baseline model was developed to simulate a scenario with seasonal malaria transmission and the effect of PCD and treatment of symptomatic infections on the *P. falciparum* malaria transmission in a low endemic area of the Peruvian Amazon. The model was then adjusted to simulate intervention scenarios for predicting the long term additional impact of FSAT on *P. falciparum* malaria prevalence and incidence. Model parameterization was done using data from a cohort study in a rural Amazonian community as well as published transmission parameters from previous studies in similar areas. The effect of FSAT timing and frequency, using either microscopy or a supposed field PCR, was assessed on both predicted incidence and prevalence rates.

**Results:**

The intervention model indicated that the addition of FSAT to PCD significantly reduced the predicted *P. falciparum* incidence and prevalence. The strongest reduction was observed when three consecutive FSAT were implemented at the beginning of the low transmission season, and if malaria diagnosis was done with PCR. Repeated interventions for consecutive years (10 years with microscopy or 5 years with PCR), would allow reaching near to zero incidence and prevalence rates.

**Conclusions:**

The addition of FSAT interventions to PCD may enable to reach *P. falciparum* elimination levels in low endemic areas of the Amazon Region, yet the progression rates to those levels may vary substantially according to the operational criteria used for the intervention.

**Electronic supplementary material:**

The online version of this article (doi:10.1186/s13071-015-0868-4) contains supplementary material, which is available to authorized users.

## Background

Despite several decades of intense control efforts, malaria remains an important public health problem in Peru, especially in the Amazon region where most (85%) of the malaria cases occur, mainly due to *P. vivax* [1]. Increased financial support from international donors, e.g., the Global Fund-PAMAFRO Project (2005–2010), allowed for the scaling-up of comprehensive malaria control strategies such as the use of artemisinin-based combination therapy (ACT) and the distribution of long-lasting insecticidal mosquito nets (LLINs) [2]. During this period, malaria declined drastically in Peru from 87,805 reported clinical cases in 2005 to 29,355 and 23,075 cases in 2010 and 2011, respectively. Since 2011, following the reduction of financial support allocated to malaria control activities, malaria incidence started to increase again with a doubling of clinical cases in 2013, highlighting the potential risk of rapid malaria resurgence in the Amazon Region [1].

In contrast to the classical statement that the development of acquired malaria immunity requires long-term exposure and intense transmission, recent studies in endemic countries of South America have reported a large proportion of asymptomatic malaria infections [3-12] suggesting that clinical immunity may develop as well in low-transmission settings of the subcontinent. The wider use of molecular diagnostics in epidemiological studies allows for a better knowledge on the extent of these asymptomatic infections, thus uncovering a “hidden” human infectious reservoir likely to contribute in maintaining transmission in endemic areas [9,13]. Indeed, cross-sectional surveys conducted in rural communities surrounding Iquitos city in Peru reported prevalence of asymptomatic parasite carriers ranging from 5 to 14%, mostly detectable only by PCR (sub-microscopic infections) [3-5].

National malaria surveillance in the Amazon Region relies on passive case detection (PCD) with microscopy [14]. Patients presenting with fever or any other symptoms compatible with malaria are systematically tested by microscopy at peripheral health facilities and treated following national guidelines. However, the major limitation of PCD is that asymptomatic individuals do not seek medical care and therefore remain undetected and untreated [4,15], extending the period of parasite carriage [16]. Other described limitations of PCD include the quality of microscopy-based diagnosis [17], the access to health services with available microscopy [18], and social and cultural factors that influence treatment seeking behaviour at community level [19].

Active case detection (ACD) has recently been recommended in low transmission and malaria-elimination settings to overcome the inherent limitations of PCD and target the asymptomatic parasite reservoir [20]. According to the World Health Organization (WHO), ACD is defined as the detection of malaria infections through household visits in population groups that are considered to be at risk. In the Peruvian Amazon, focal screening and treatment (FSAT), has recently been introduced by the National Malaria Control Program (NMCP) to target malaria hotspots and further decrease the malaria burden. Malaria hotspots are well-delimited villages in rural areas or neighborhoods in periurban areas in which the cumulative malaria incidence over the past 3–5 years is the highest. These hotspots represent ~10-20% of the total malaria reporting-localities and account for more than 80% of the total cases in the Region. The intervention consists of systematic finger prick blood sampling of the whole population (100–1000 inhabitants) during the period of highest incidence (usually between February and May) and the treatment of all microscopically positive infections, irrespective of the presence of symptoms [14]. Following the national treatment guidelines, *P. falciparum* infections are treated with an ACT -mefloquine and artesunate (MQ-AS)-, and *P. vivax* infections with chloroquine (CQ) and primaquine (PQ) [14]. However, due to the lack of evidence-based guidelines [21,22], the criteria and procedures to implement FSAT interventions in the Amazon Region have often been arbitrarily decided and are subject to annual variations in logistical and financial resources.

Mathematical models of various levels of complexity have been used for understanding malaria transmission dynamics and informing decision-making in malaria control and elimination efforts [23-26]. Here, we used a model to assess the long term impact of FSAT on the *P. falciparum* malaria transmission intensity (MIT) in a low endemic area of the Peruvian Amazon Region. Retrospective data from a cohort study in a rural community of the Peruvian Amazon were used to estimate infection rates and a time-variant seasonal vector parameter, and then to evaluate the model. Other malaria-transmission parameters were obtained from previously published studies in the Amazon region. Eventually, the model was used to predict the potential reduction in malaria transmission following the addition of FSAT to the ongoing PCD, allowing for a simulation of the effect size according to the diagnostic tools used, as well as the timing and frequency of FSAT interventions.

## Methods

### Baseline model

**Figure 1:**
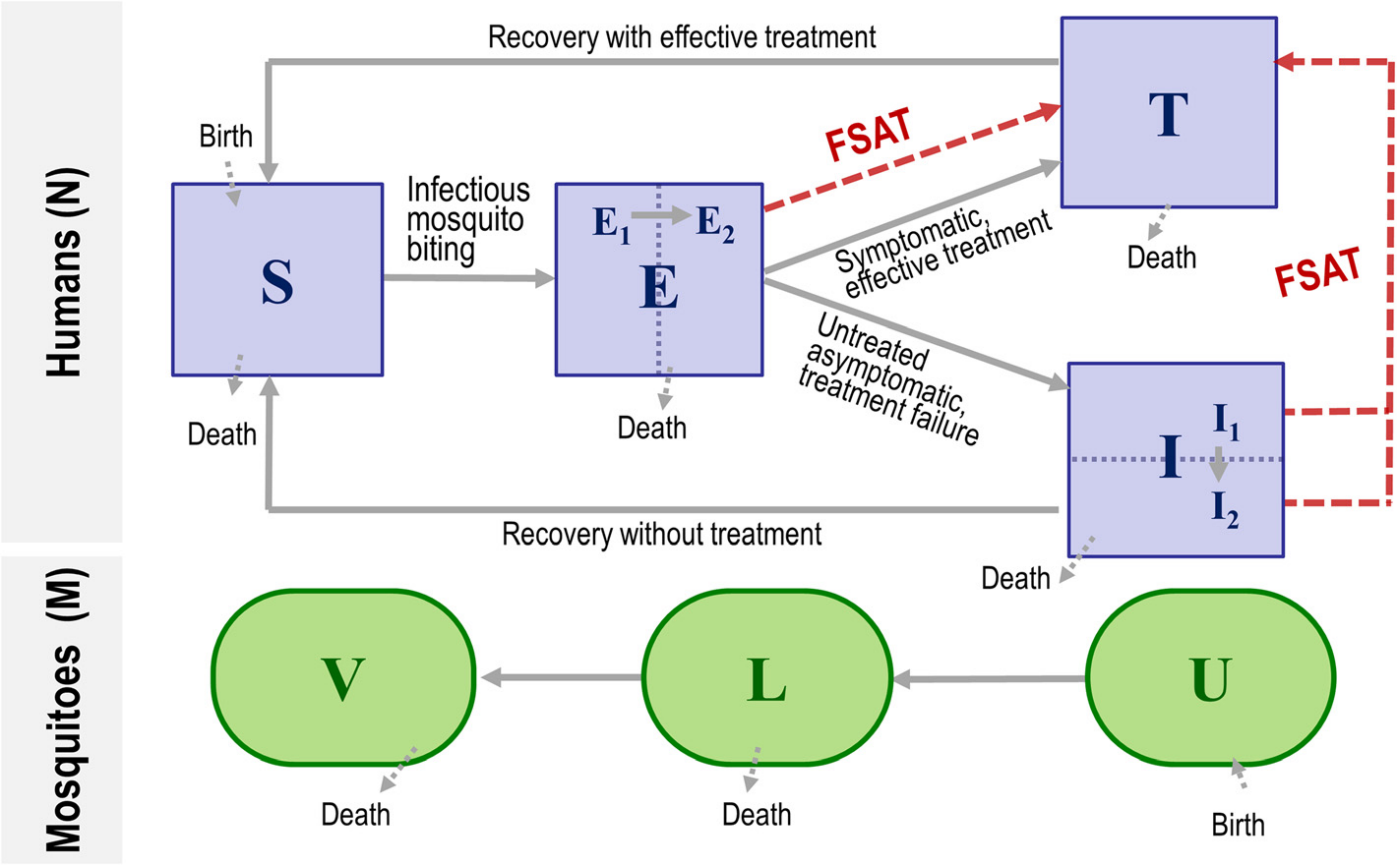
Schematic diagram of the human and vector model states. The human population (N) is divided into 4 compartments: susceptible (S), infected latent (E), infectious treated (T) and infectious untreated (Ι) individuals. The latent period has 2 sub-states: latent which have not yet developed asexual blood-stage parasites (E_1_) and latent with asexual blood-stage parasites (E_2_). Before becoming recovered, individuals in I compartment need to pass through 2 sub-states: those who have not yet (Ι_1_) and those who have spontaneously cleared the asexual blood stage parasites (Ι_2_). The mosquito population (M) is divided into 3 compartments: uninfected (U), infected latent (L) and infectious (V) mosquitoes. The addition of FSAT to PCD allows for the detection and treatment of individuals in E_2_, I_1_ and I_2_ compartments.

An extension of the Anderson-and-May’s malaria transmission model [27] and the corresponding differential equations was used to simulate a scenario where seasonal malaria transmission remains at similar levels each year within a restricted human population with ongoing PCD and treatment of symptomatic *P. falciparum* infections (Figure 1 and Additional file 1: Figure S1). The deterministic model allows the human population (N) to move between four different states (compartments), and the mosquito population (M) between three states. Individuals initially start in a susceptible state (S) and become infected at a rate that is determined by the mosquito density (*m*), the human feeding rate per mosquito *(a*), the infectiousness of the mosquito population (V/M), and the susceptibility of an individual after being bitten by an infectious mosquito (*b*). Individuals progress to the latent state (E), from which they develop gametocytes in the blood and become infectious after a latent period (*l*). Two consecutive sub-states are distinguished in infected latent individuals (E_1_ and E_2_) thus dividing the latent period into two time intervals: between sporozoite inoculation and development of detectable asexual stage parasites in blood (*l*_*1*_), and between asexual parasite infection and development of gametocytes (*l*_*2*_). Then, the number of individuals who move from E_1_ to E_2_ at time *t* is equal to the number of individuals who entered E_1_ at time *t-l*_*1*_ adjusted by the factor e^-l1*h^, which incorporates the human mortality rate (*h*) and the average time interval for developing asexual blood parasites (*l*_*1*_). On the other hand, infected individuals leave sub-state E_2_ at a rate (*1/l*_*2*_) which is the reciprocal of average duration of the gametocytogenesis process.

While a constant fraction (*y*) of symptomatic infections of all newly infected individuals is routinely detected by PCD and treated with an ACT of known effectiveness (*ε*) within the treated state (T), the remaining fraction (1-y*ε), including asymptomatic individuals and previously treated individuals with parasitological treatment failure, remains infectious in an untreated state (I) until the “spontaneous cure” of the disease (i.e. clearance of all parasite forms) unless they are actively detected and treated. Once recovering from compartments T and I, individuals return to the susceptible state (S). While recovery rate for treated individuals (*1/r*_*1*_) is the reciprocal of average human infectious period (i.e. length of time gametocytes remain in the bloodstream) with effective treatment, recovery for untreated individuals requires they pass through two sub-states before becoming recovered: those who have not yet (I_1_) and those who have spontaneously cleared the asexual blood stage parasites (I_2_). Then, the rate of movement of individuals from I_1_ to I_2_ (*1/r*_*2a*_), and from I_2_ to S (*1/r*_*2b*_) are the reciprocal of the average time for the spontaneous clearance of asexual parasites from blood (*r*_*2a*_), and the remaining average infectious period after the clearance of blood asexual parasites (*r*_*2b*_), respectively in untreated individuals.

Regarding the mosquito population (M), the model includes an initial susceptible state (U) for all mosquitoes, becoming infected at a rate determined by the following three parameters: human feeding rate per mosquito (*a*), fraction of infectious individuals in the human population ((T + I)/N), and the mosquito susceptibility to infection after biting an infectious individual (*c*). Infected mosquitoes remain in a latent state (L) for a specific time period before developing sporozoites and becoming infectious (V). The estimation of surviving mosquitoes by the end of the latent period requires an adjustment by the factor *e*^-g*n^ which incorporates a constant mosquito mortality rate (*g*) and the average mosquito latent period (*n*). The model assumes that mosquitoes remain infectious until they die.

The main assumptions of this model are the following: i) human and mosquito populations remain constant (number of births and deaths are equal at any time); ii) malaria-attributable deaths are not explicitly incorporated (malaria mortality rate is similar to general mortality rate); iii) no individual differences in transmission parameters between subjects; iv) super-infections do not occur [28]; v) all infected individuals develop mature gametocytes immediately after they leave the latent sub-state E2; vi) any gametocytemia represents potential for transmission; and vii) there is no prophylactic effect of the drugs post recovery.

### Retrospective data on malaria cohort study

The model was fine-tuned by using retrospective data from a cohort study carried out between 2003 and 2008 in four rural communities of San Juan district, south of Iquitos city in the Peruvian Amazon [4,28,29]. Malaria transmission in this area typically starts in January and finishes in July with a peak of clinical malaria cases between February and March; *P. vivax* and *P. falciparum* infections occurring at a ratio 4:1. Previous reports from this area indicated that more than two thirds of *P. falciparum* infections were asymptomatic with low parasite density [3,4]. *Anopheles darlingi* is the major malaria vector with a strong anthropophilic behavior [30,31]. The initial cross-sectional survey conducted in April 2003 among cohort participants showed that most of them reported sleeping under bed nets, although none of them mentioned the use of insecticide treated nets or repellents in their homes [4]. From 2004 to 2008, biannual community screening campaigns were carried out (beginning-end of each malaria season), and in between, weekly ACD for *P. falciparum* infections was conducted, sampling each month a new group of individuals living within a 100-m radius of houses where at least one *P. falciparum* infection was detected during the previous month (index houses), either through the surveys, ACD or the routine PCD at health facilities [4]. Since high coverage during screening campaigns was achieved only in Ninarumi community, local model parameters were estimated from this community only, and taking into account malariometric indices from the year 2004 only, which was the first full year of ACD in the cohort study. The prevalence of *P. falciparum* infections by microscopy in Ninarumi was measured at 6.5% and 1.1%, respectively in January and August 2004 (Additional file 2: Table S1); and during that year a total of 139 new *P. falciparum* infections were detected (0.28 infections/person/year), 91 (65.4%) of them being symptomatic either at the time of sampling or during the following week. Of the additional 48 infections which remained asymptomatic during the follow-up, 19 were confirmed only by retrospective PCR analysis on blood samples collected on filter paper from individuals during weekly ACDs. The detailed procedures for the PCR analysis are described elsewhere [29]. All individuals enrolled in the cohort study gave signed informed consent or assent. Ethical clearance was received from New York University (NYU Institutional Review Board [IRB] approval no. 08–982), the University of Alabama at Birmingham, and the Peruvian Ministry of Health and National Institutes of Health Internal Ethical Review Boards [29].

### Transmission parameter values

Considering days as the time unit, the model parameterization was done in several steps, i.e. using first the available cohort data for the estimation of important local parameters, and in a second step, using published data to improve the first step estimates or to set values for the remaining transmission parameters. A systematic literature search was undertaken in different databases (i.e. PubMed, EMBASE and SciELO) prioritizing published studies on malaria in the Amazon Region in English and Spanish language, especially those from the Iquitos area when available. Additional searches were conducted in Google and through reference tracking. Third, when parameters could not be identified in previous steps, they were estimated by fitting the model to the observed clinical malaria incidence in Ninarumi. Table 1 summarizes key parameter values used in the baseline model (see Additional file 3: Text S1 for a detailed description).

**Table 1:** **Model parameters**

**Parameters**		**Symbol**	**Value**	**Source**
**Baseline model parameters**				
	Mosquito density (ratio female mosquitoes/humans)	*m*	75	Assumed value within published range
	Human feeding rate, bites per mosquito per day	*a*	0.035 - 0.32	Monthly estimations from fitting cohort data; values within published range
			Mean: 0.088	
	Susceptibility of an individual to infection after being bitten by an infectious mosquito	*b*	0.05	Estimation using cohort and published data
	Mosquito susceptibility to infection after biting an infectious individual	*c*	0.41	Published data
	Duration of human latent period for Pf* in d]’ cays *(l = l*_*1*_ *+ l*_*2*_ *)*	*l*	21	Published data
	Duration from sporozoite inoculation to development of blood asexual parasites in days	*l*_*1*_	11	Published data
	Duration from asexual blood stage infection to development of gametocytes in days	*l*_*2*_	10	Published data
	Duration of mosquito latent period in days	*n*	11	Published data
	Daily human mortality rate	*h*	0.00005	Published data
	Daily mosquito mortality rate	*g*	0.16	Published data
	Fraction of symptomatic infections	*y*	0.65	Cohort data
	Treatment effectiveness	*ε*	0.98	Published data
	Duration of infectious period in treated humans with ACT (in days)	*r*_*1*_	14	Published data
	Duration of infectious period in untreated humans in days *(r*_*2*_ *= r*_*2a*_ *+ r*_*2b*_ *)*	*r*_*2*_	200	Published data
	Duration of presence of blood asexual parasites in untreated humans in days	*r*_*2a*_	80	Published data
	Duration of infectious period in untreated humans after clearance of blood asexual parasites in days	*r*_*2b*_	120	Published data
**FSAT parameters**				
	Coverage of FSAT	Δ	1	Assumption
	Duration of FSAT in days	*v*	1	Assumption
	Sensitivity of microscopy for detecting Pf* asexual stages	*s*_*1a*_	0.5	Published data
	Sensitivity of microscopy for detecting Pf*gametocytes	*s*_*1g*_	0.1	Published data
	Sensitivity of highly sensitive diagnostic tests for detecting asexual stages	*s*_*2a*_	0.95	Assumption
	Sensitivity of highly sensitive diagnostic tests for detecting gametocytes	*s*_*2g*_	0.8	Assumption

*Pf = P. falciparum.

### FSAT model and assessment of interventions

In the scenario with FSAT interventions, the baseline model was improved by allowing movements from sub-states E_2_, I_1_ and I_2_ to the treated state (T) (red dotted lines in Figure 1), at specific rates that are determined by the coverage (∆), the reciprocal of duration of the intervention (*1/v*), the sensitivity of the diagnostic test used for malaria screening (*s*), and the effectiveness of treatment in confirmed infections (*ε*).

After setting initial conditions for human and mosquito compartments based on the average observed prevalence in the 2004 Ninarumi surveys and the reported *A. darlingi* sporozoite rates in the area [30], the baseline model was run until reaching steady levels (baseline scenario), indicated by the stable cumulative number of *P. falciparum* infections recorded for 20 more simulated years. Then, the model was run for an additional year (year 0) after which interventions based on FSAT (intervention scenario) were added to the routine PCD (baseline scenario). A first intervention scenario was modeled with the following assumptions: one FSAT during the peak of the high transmission season (only in February of year 1); FSAT covers 100% of population (*∆* = 1) in one day (*v* = 1); microscopy used in FSAT allows for the detection of 50% of asexual blood-stage infections by *P. falciparum* (sub-states E_2_ and I_1_), and 10% of infections with only sexual stages (gametocytes) in blood (sub-state I_2_) [32]; and all detected *P. falciparum* infections are treated with a 98% effectiveness (*ε* = 0.98).

Additional intervention scenarios were modeled to assess the impact of different operational criteria for FSAT implemented during only one year (i.e. timing, number and time intervals between consecutive interventions) on the predicted cumulative incidence and prevalence of *P. falciparum* infections. Besides microscopy, a field PCR was also considered as diagnostic test during malaria screening, assuming sensitivities of 95%, 95% and 80% for detecting infections in sub-states E_2_, I_1_, and I_2_, respectively. The predicted cumulative incidence (i.e. cumulative number of new symptomatic and asymptomatic infections) was recorded over one-year and five-year periods from the start of the first FSAT in each intervention scenario, and was compared with the cumulative incidence found with the baseline PCD scenario. The predicted impact of each intervention was determined as the percentage difference between the cumulative number of new *P. falciparum* infections with baseline and with the respective intervention scenarios (cumulative number of infections averted). In addition, minimum and maximum predicted prevalence were determined during both the first and the fifth year after the onset of FSAT interventions. Finally, operational criteria for implementing FSAT interventions during only one year which had the strongest impact were used again to assess the effect of the same interventions if implemented during several consecutive years.

Malaria transmission scenarios were simulated in R software version 2.15.3 (function “dede” in deSolve package) allowing to solve delay differential equations to take into account latent periods for infected individuals and mosquitoes before being infectious (see R code for baseline model in Additional file 4: Text S2) [33]. Baseline model performance was examined by comparing predicted and observed *P. falciparum* incidence and prevalence from the cohort study. A Wilcoxon matched-pairs signed-ranks test was used to test differences between observed and predicted monthly incidence, with p < 0.05 indicating statistical significance. Different initial values for the *A. darlingi* sporozoite rate (V/M) were tested and did not affect the results. Moreover, a one-at-a-time sensitivity analysis was conducted to determine the effect of the following parameters on the baseline model predictions [34]: the mosquito density (*m*), the human feeding rate (*a*), the fraction of symptomatic infections (*y*), and the treatment effectiveness (*ε*). Changes (increase and decrease) of 20% in parameter values were considered in the analysis. Finally, only human parameters (*y*, *ε*) were used later to assess their impact on the predictions of the intervention scenarios with FSAT.

## Results

**Figure 2:**
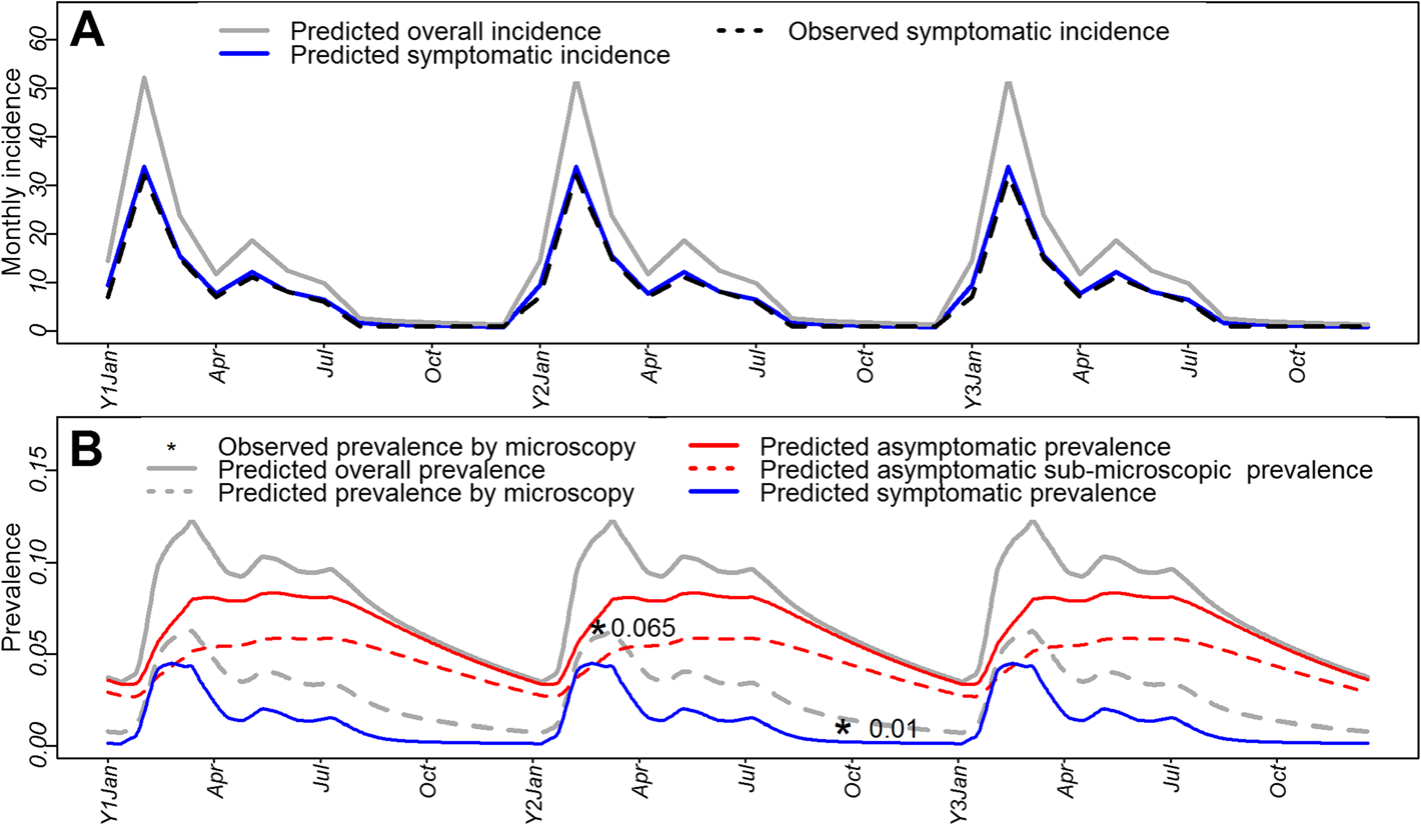
Comparison between observed and baseline model-predicted *P. falciparum* malariometric measures in Ninarumi village. Curves with data from Ninarumi in 2004 were repeated for three consecutive years: **A)** observed and predicted *P. falciparum* monthly new *P. falciparum* infections (monthly incidence); **B)** observed and predicted *P. falciparum* prevalence.

Baseline model predictions largely reflected *P. falciparum* incidence rate in Ninarumi in 2004 (Figure 2A), with no statistically significant difference between observed and predicted monthly incidence of symptomatic *P. falciparum* infections. While Ninarumi reported 91 symptomatic and 38 asymptomatic *P. falciparum* infections (total = 139 infections) in 2004, the baseline model predicted 99 symptomatic and 43 asymptomatic infections (total = 152 infections). Furthermore, predicted prevalence curves accurately reflected the seasonal dynamics of malaria infections (Figure 2B), with high transmission season (HTS) between January and July, and low transmission season (LTS) between August and December. According to the baseline model predictions, the maximum overall and microscopically determined prevalence during HTS were 12.3% and 6.2%, respectively; while the corresponding minimum values during LTS were 3.4% and 0.7%, respectively. Therefore, predicted and observed prevalence by microscopy also correlated well in Ninarumi in 2004. Furthermore, the proportion of detected infections by microscopy among the total predicted infections was significantly higher in the peak of HTS (~50%) than in LTS (~10-20%). Similarly, the proportion of asymptomatic infections among the total predicted infections varied according to the season, from a minimum of 58% during the peak of HTS to a maximum of 96% in the middle of LTS.

**Figure 3:**
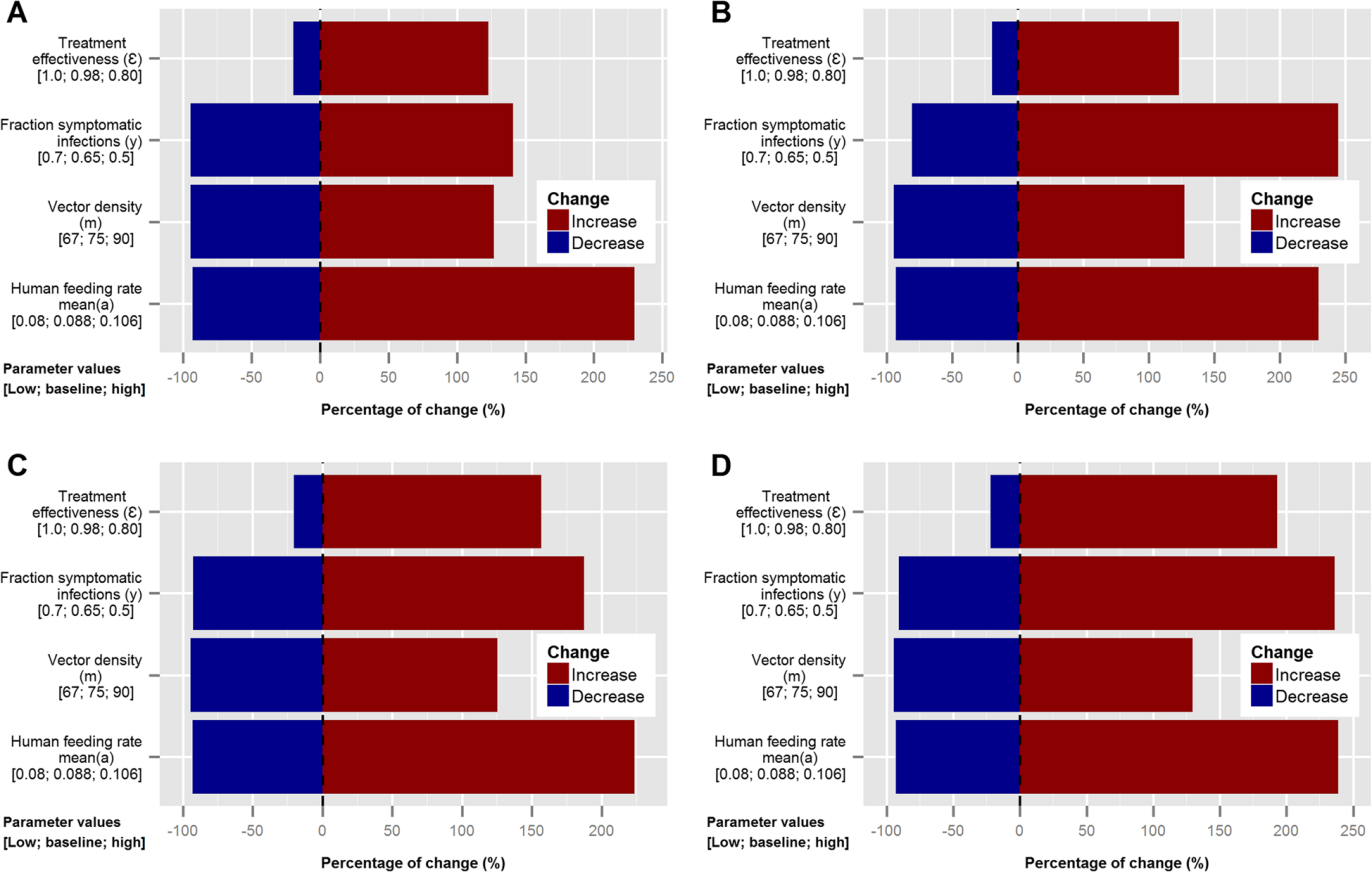
Sensitivity analysis for the baseline model. Changes in: **A)** annual overall incidence (baseline model prediction: 152 infections); **B)** annual asymptomatic incidence (baseline: 53 infections); **C)** maximum prevalence during the year (baseline: 12.3%); **D)** minimum prevalence during the year (baseline: 3.5%). Results are expressed in percentage of change (%) in comparison with baseline model predictions.

The sensitivity analysis for the baseline model (Figure 3) showed that with values above 0.7 for the fraction of symptomatic infections (*y*), malaria transmission decreased progressively until almost undetectable levels. In addition, seasonal malaria transmission persisted only when mosquito density (m) and mean expected number of bites on humans per mosquito (*a*) are higher than 67 and 0.080, respectively. Increasing monthly values of human feeding rate (*a*) by 20% had by far the strongest effect on the annual incidence rate, with more than three times the baseline number of *P. falciparum* infections (~225% more infections) (Figure 3A). On the other hand, a decrease from 0.65 to 0.5 in the fraction of symptomatic infections (*y*) had the highest impact on the predicted incidence of asymptomatic infections (increase of baseline incidence by ~250%) (Figure 3B). Moreover, the same changes in both parameters (i.e. human feeding rate and fraction of symptomatic infections) had similar effects on the predicted prevalence, almost triplicating the maximum and minimum prevalence in HTS and LTS, respectively (Figure 3C and D). Furthermore, a reduction of the treatment effectiveness from 0.98 (baseline value) to 0.80 would increase the incidence and prevalence by more than 100% and 150%, respectively.

**Figure 4:**
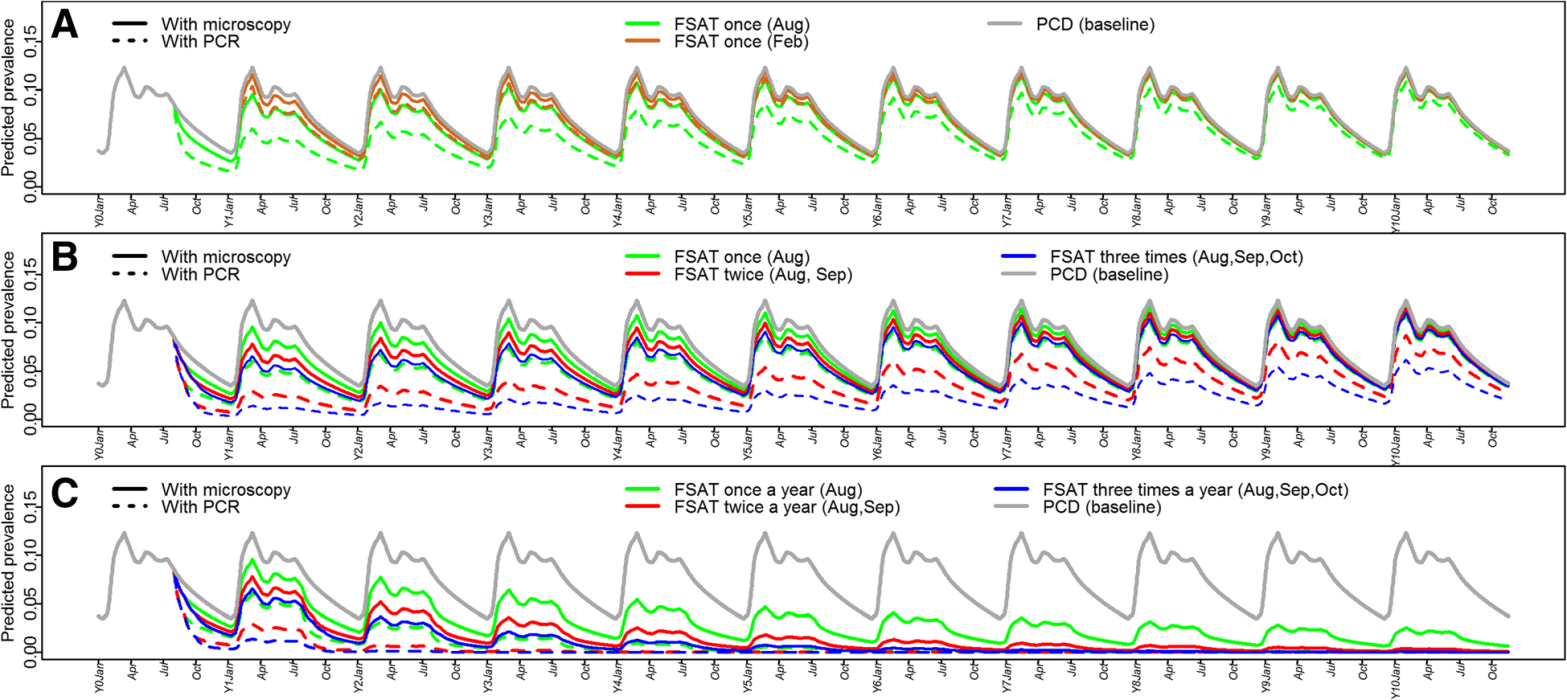
Model-predicted *P. falciparum* prevalence curves after the addition of different FSAT interventions to the baseline PCD. **A)** one FSAT at beginning of LTS only in year 0 (Y0) and in HTS only in year 1 (Y1), using microscopy or PCR; **B)** one to three successive FSAT starting at beginning of LTS only in Y0, with microscopy or PCR; **C)** one to three successive FSAT starting at beginning of LTS for consecutive ten years (from Y0 to Y10), with microscopy or PCR.

Table S2 shows the predicted impact (after 1 and 5 years) on the *P. falciparum* incidence after the addition of different FSAT interventions according to timing and number of interventions. Conducting one FSAT intervention with microscopy alone between July and September during only one year would have a larger impact on the *P. falciparum* incidence than conducting it during any other month, with a reduction of about 20% and 15%, respectively at 1- and 5 years post intervention. However, the magnitude of this effect would be twice as high if PCR is used instead of microscopy, reaching reductions of more than 40% and 30% respectively at 1- and 5 years after intervention. Similarly, the impact on *P. falciparum* prevalence was the highest when FSAT was added in LTS (in August), instead of in HTS (in February) during only one year (Figure 4A), with a significant reduction in the maximum prevalence reached during the next HTS (p < 0.05), i.e. from 12.3% (baseline prevalence) to 9.6% (using microscopy) or to 6.0% (if PCR is used).

The strongest decrease in *P. falciparum* malaria incidence for interventions implemented during only one year was observed with three consecutive FSAT starting at the beginning of LTS and separated by time intervals of 7 to 60 days (Additional file 5: Table S2), resulting in reductions of more than 40% and 30%, respectively after 1 and 5 years when using microscopy, and of more than 80% and 75%, respectively when using PCR. The impact of the number of consecutive FSAT at the start of LTS (one to three FSAT during only one year, separated by intervals of 30 days) on the predicted *P. falciparum* prevalence is shown in the Figure 4B. While three consecutive FSATs using PCR would have the largest effect on the predicted prevalence, one FSAT alone using PCR would result in a similar reduction compared to three consecutive FSATs using microscopy. In all simulations including PCR, annual *P. falciparum* incidence and prevalence would return slowly to the initial levels after 10 years, if FSAT interventions are implemented only during one year.

**Figure 5:**
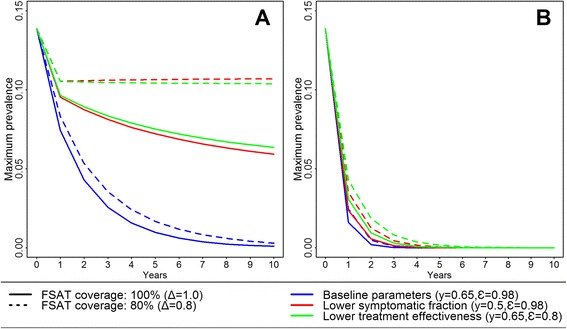
Long-term impact of changes in the fraction of symptomatic infections and the treatment effectiveness on the maximum predicted *P. falciparum* prevalence after the onset of annual FSAT interventions (three successive FSAT at beginning of LTS): **A)** FSAT interventions using microscopy for malaria screening; **B)** FSAT interventions using PCR for malaria screening.

Figure 4C shows a progressive reduction of the predicted prevalence with annually repeated FSAT interventions starting at the beginning of LTS every year. All repeated interventions would reach the near-zero prevalence, however the rate of decrease largely depends on the number of monthly FSAT per year and the diagnosis test used for malaria detection. Indeed, while three-monthly FSATs using microscopy would require approximately 10 years to reach the zero level prevalence; this could be reached with three monthly FSATs using PCR for four consecutive years. However, a decrease in the fraction of symptomatic infections from 0.65 to 0.5, or a decrease in the treatment effectiveness from 0.98 to 0.8, would substantially reduce the rate of progression to the elimination levels with FSATs using microscopy (Figure 5A), and consequently increase the time required to reach the elimination levels to more than 20 years. Furthermore, if a reduction of FSAT coverage from 100% to 80% is added to those scenarios, *P. falciparum* prevalence would start to increase again despite of the FSAT interventions. Conversely, if annually repeated FSAT interventions are implemented using PCR, the effects of similar changes (i.e. decrease in the fraction of symptomatic infections, in the treatment effectiveness, or in the FSAT coverage) on the *P. falciparum* prevalence would be smaller, hence delaying the time to reach zero prevalence levels only by one or two years (6 or 7 years after onset of interventions) (Figure 5B).

## Discussion

Using a relatively straightforward mathematical model, our analysis showed that targeting the asymptomatic parasite reservoir with FSAT interventions, in addition to routine PCD, would significantly reduce *P. falciparum* malaria in low transmission areas of the Peruvian Amazon. The strongest reduction was observed if three consecutive FSAT were implemented annually at the beginning of low transmission season, and if malaria diagnosis was done with a highly sensitive field PCR.

Modelling the effects of interventions that target the asymptomatic malaria reservoir requires a good knowledge of the burden of asymptomatic infections and their transmission dynamics. Cross-sectional surveys in the Peruvian Amazon reported that more than two thirds of malaria infections were asymptomatic [3,4]; however these studies did not take into account the incubation period for some of them, hence the potential development of symptoms at a later stage [9]. Therefore data from the cohort study conducting in peri-Iquitos communities including weekly monitoring of symptoms for one month of all infected individuals, enabled to overcome the limitations of previous survey results. Indeed, an important proportion of *P. falciparum* infections which were asymptomatic at the time of detection (either by microscopy or by retrospective PCR) developed malaria symptoms (reported and/or measured fever) after one week of follow-up [4], resulting in only 35% of asymptomatic infections among all confirmed cases in the Ninarumi community in 2004. Another study conducted in the Brazilian Amazon in 2004–2005 found similar results after combining PCD and ACD over a 14-month period, i.e. a total of 326 malaria infections were identified of which 96 (29.4%) were asymptomatic– a much lower proportion than reported in any of the cross-sectional surveys carried out during the same period [35].

The cohort study also allowed for an accurate measurement of seasonal variations in the incidence of symptomatic and asymptomatic *P. falciparum* infections in the area, and these seasonal dynamics were well reproduced by the baseline model. In addition, the model predicted not only a 2–3 times higher prevalence of infections during the peak of HTS than during the LTS, but also temporal differences in the proportion of asymptomatic infections, which accounted for about 60% of all prevalent infections during the peak of HTS while they represented almost all infections in the middle of LTS. Moreover, the proportion of prevalent infections detectable by microscopy (“patent infections”) varied also with season, i.e. from almost 50% (6.2% of a total prevalence of 12.3%) in the HTS to 20% (0.7% of a total prevalence of 3.4%) in the LTS. Those predictions suggest that in areas of low and seasonal transmission such as the Peruvian Amazon, non-detectable asymptomatic *P. falciparum* infections are able to persist during the LTS being potentially infectious to mosquitoes emerging after the start of the rains, as previously described in other areas with markedly seasonal transmission [36,37].

Since symptomatic *P. falciparum* infections are more likely to be identified and treated during PCD, and the duration of treated infections is considerably shorter than untreated infections; it is anticipated that potential changes in the fraction of symptomatic infections as well as in the effectiveness of case management, may significantly impact the malaria prevalence as shown by the sensitivity analysis. Indeed, simulations showed that a decrease in the fraction of symptomatic infections from 0.65 to 0.5 or a decrease in the treatment effectiveness from 0.98 to 0.8 resulted in similar effects as changes in entomological parameters (ratio female mosquitoes to humans from 75 to 90, human feeding rate by 20%) which are known as being important determinants for the vectorial capacity and the malaria transmission [38]. Asymptomatic infections are becoming increasingly common at low transmission levels [3-12,21], enlarging the human infectious reservoir which will enable onward transmission. On the other hand, treatment effectiveness can decrease due to the spread of drug-resistant strains of *P. falciparum* parasites and the lack of systems that assure the quality of both the antimalarial drugs and the case management procedures (e.g. poor prescribing practices by health workers, low patient adherence rates to treatment regimens) [22,39].

In contrast to the current strategy of the National Malaria Control Program consisting of adding FSAT with microscopy to PCD during HTS in prioritized localities of the Peruvian Amazon [14], our simulations showed that conducting a single FSAT at the beginning of LTS would not only result in a greater reduction in *P. falciparum* incidence and prevalence in the short term, but also decrease the progression rates of these indicators to the initial levels. Furthermore, the effect of FSAT would be maximal if three successive FSAT at time intervals of 7–60 days, would be implemented at the start of the LTS. Kern *et al.* found a similar impact on the *P. falciparum* transmission with a single FSAT with microscopy during the dry season using a seasonally-forced malaria model which incorporated periodically mosquito birth rates through a sinusoidal function and different levels of infectiousness for gametocyte carriers; however, while in areas of simulated low disease burden (entomological inoculation rates (EIR) < 10 infective bites/person/year) such as the Peruvian Amazon, the effect was sustained for over three years after the intervention, in areas of high endemicity (EIR > 200) the incidence rates of infection returned to their initial level in the subsequent year [40]. Moreover, the study also indicated that three FSAT scheduled at monthly intervals starting in the dry season had the greatest positive impact on transmission. Successive FSATs would not only allow for identifying infected individuals who were in a latent state at the time of the previous FSAT, but also would increase the coverage of the intervention in real-life situations by including individuals who were absent or not eligible (illness, pregnancy) during the previous FSAT. The relatively wide time interval (i.e. 7–60 days) for a subsequent intervention suggested by our model would facilitate the planning and logistics of mobile teams in charge of FSAT in the targeted communities.

Beside timing and frequency of a given intervention, the other important determinant of impact is the duration (number of years) the intervention needs to be applied to reach sustainable elimination levels. Our model suggested that three successive FSAT with microscopy for ten consecutive years, would result in a sustained declining *P. falciparum* prevalence and incidence and eventually reach elimination levels. Long-term effects of FSAT has been previously described by Griffin *et al.* using a complex modelling approach (including immunity, super-infections, and heterogeneity in transmission intensity) fed with published transmission parameters and local epidemiological data of different endemic areas of Africa [41]. The results showed that in low-to-moderate transmission settings, 80% coverage of annual single round of FSAT using rapid diagnostic tests (RDTs) over 15 years, together with 80% coverage of insecticide treated nets (ITNs), would reduce the absolute parasite prevalence by 5–12%. On the other hand, in high transmission areas, the simulated impact of FSAT alone was marginal, the ultimate outcomes strongly depending on the initial transmission level, the frequency and duration of the intervention and the coverage of vector control (i.e. either ITN or indoor residual spraying) [41].

As a consequence of increased use of molecular tests for detecting submicroscopic malaria infections in both low and high-malaria transmission settings, as well as of recent studies reporting failures of FSAT to reduce transmission when using microscopy or RDTs [42], it has been suggested that PCR based strategies need to be deployed if such interventions are to be successful [43,44]. Our model simulations assessed the impact of FSAT interventions using an assumed field PCR with high sensitivity, under the assumption that any level of gametocyte density was potentially infectious [45]. Repeated FSAT interventions with PCR for several consecutive years would not only have a stronger impact on *P. falciparum* malaria transmission (halving time to reach elimination levels), but would also enable to sustain the impact despite eventual reductions in the proportion of symptomatic infections, treatment effectiveness and/or coverage of FSAT. Although these results support the importance of detecting and treating sub-microscopic infections at point of care for successful elimination strategies [22,46], implementing FSAT using PCR as routine strategy in prioritized localities of the Peruvian Amazon may not be feasible since current PCR tests are resource-intensive, time-consuming and expensive [47]. Recently, some studies have shown high sensitivity of non-conventional molecular tests based on loop-mediated isothermal amplification (LAMP) with no requirement for specialist staff and expensive equipment [48]; these molecular tests need to be optimized quickly to be used in the field for species-specific identification of *Plasmodium* parasites.

Like with other mathematical models for malaria transmission, our predictions should be interpreted taking into account the underlying assumptions. Some assumptions were intended to simplify the complex malaria dynamics (e.g. constant mosquito and human populations, no individual differences in transmission parameters between subjects), while others attempted to fill the knowledge gap in the *P. falciparum* malaria transmission cycle (e.g. the development of mature gametocytes in all infected individuals, potential for transmission of any infectious individual independently of its gametocyte density in blood). Further research is still needed to define to what extend asymptomatic and submicroscopic malaria infections contribute to the infectious reservoir [49]; this information would considerably improve our model. In addition, after the development of a more complex model for *P. vivax* transmission using data of a recent cohort carried in the Peruvian Amazon [12], it will help to further fine-tune the model and define operational criteria for FSAT interventions to maximize the impact of the intervention on both species.

## Conclusions

Our model suggested that FSAT combined to PCD enables to reach elimination levels in an area of low transmission of the Amazon Region, but the progression rates to those levels vary substantially according to the operational criteria used for the intervention (i.e., timing, number, coverage, and duration of FSAT) and the sensitivity of the diagnostic test used for malaria screening. Despite its limitations, the modelling approach can contribute to design new field studies in the Peruvian Amazon to assess the effectiveness of FSAT and other strategies to further decrease malaria transmission. Even before easy-to-use molecular tests will be available at the point of care, as well as reliable evidence of the effectiveness of FSAT, the current strategy applied by the NMCP in Peru should be revised in the light of our results in order to be more cost effective. Programmatic FSAT interventions with microscopy (minimum of two successive FSAT) can be conducted at the beginning of the LTS in areas with known high risk of *P. falciparum* malaria transmission, trying to reach the maximum population coverage (avoiding areas with highly mobile populations). Alternatively to reach the areas of mobile populations, resources could be invested to implement a “smart surveillance system” based on reactive case detection guided by information from the PCD.

## Additional files

Additional file 1: Figure S1. Schematic diagram of the human and vector model states, including all transmission parameters. The total human population (N) is divided into 4 compartments: susceptible (S), infected latent (E), infectious treated (T) and infectious untreated (Ι) individuals. The latent period has two sub-states: latent which have not yet developed asexual blood-stage parasites (E_1_) and latent with asexual blood-stage parasites (E_2_). Before becoming recovered, individuals in Ι compartment need to pass through two sub-states: those who have not yet (Ι_1_) and those who have spontaneously cleared the asexual blood stage parasites (Ι_2_). The total mosquito population (M) is divided into three compartments: uninfected (U), infected latent (L) and infectious (V) mosquitoes. The addition of FSAT to PCD allows for the detection and treatment of individuals in E_2_, Ι_1_ and Ι_2_ compartments. Additional file 2: Table S1.
*P. falciparum* malaria prevalence and incidence rate in Ninarumi in 2004. Additional file 3: Text S1. Description of transmission parameters for baseline model. Additional file 4: Text S2. R-Code for the baseline model. Additional file 5: Table S2. One and five-year predicted cumulative number of *P. falciparum* infections averted after the addition of FSAT interventions to PCD at different timing, using either microscopy or PCR.
