# Supplementary figures and images for: Modelling the potential of focal screening and treatment as elimination strategy for Plasmodium falciparum malaria in the Peruvian Amazon Region
Source: Parasit Vectors. 2015 May 7;8:261. doi: 10.1186/s13071-015-0868-4 (PMC4429469; doi:10.1186/s13071-015-0868-4)

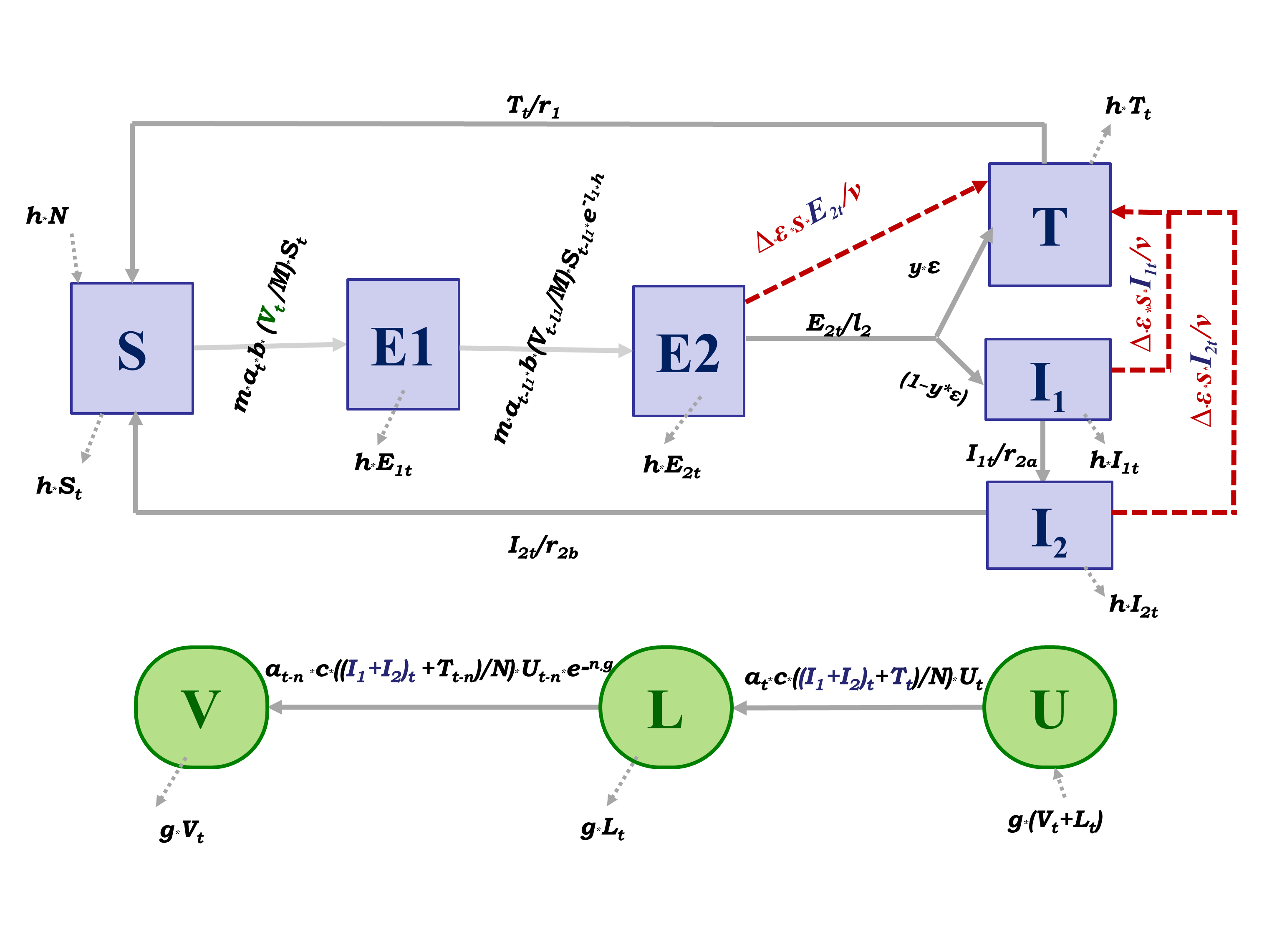

Supplement: Additional file 1: Figure S1. — Schematic diagram of the human and vector model states, including all transmission parameters. The total human population (N) is divided into 4 compartments: susceptible (S), infected latent (E), infectious treated (T) and infectious untreated (Ι) individuals. The latent period has two sub-states: latent which have not yet developed asexual blood-stage parasites (E1) and latent with asexual blood-stage parasites (E2). Before becoming recovered, individuals in Ι compartment need to pass through two sub-states: those who have not yet (Ι1) and those who have spontaneously cleared the asexual blood stage parasites (Ι2). The total mosquito population (M) is divided into three compartments: uninfected (U), infected latent (L) and infectious (V) mosquitoes. The addition of FSAT to PCD allows for the detection and treatment of individuals in E2, Ι1 and Ι2 compartments. [file 13071_2015_868_MOESM1_ESM.tif]
