# Supplementary material for: Modelling the potential of focal screening and treatment as elimination strategy for Plasmodium falciparum malaria in the Peruvian Amazon Region
Source: Parasit Vectors. 2015 May 7;8:261. doi: 10.1186/s13071-015-0868-4 (PMC4429469; doi:10.1186/s13071-015-0868-4)
Supplement: Additional file 2: Table S1. — P. falciparum malaria prevalence and incidence rate in Ninarumi in 2004. [file 13071_2015_868_MOESM2_ESM.docx]

**Table S1. *P. falciparum* malaria prevalence and incidence rate in Ninarumi in 2004**

|  | | **n** | **N** | **%** | **95% CI** |
| --- | --- | --- | --- | --- | --- |
|  |  |  |  |  |  |
| **Prevalence by microscopy** | February 2004 | 28 | 432 | **6.5** | [5.6; 7.4] |
|  | August 2004 | 3 | 269 | **1.1** | [0.3; 2.0] |
|  |  |  |  |  |  |
| **Annual incidence** | Symptomatic infections | 91 | - | **65.4** | **-** |
|  | Asymptomatic microscopically confirmed infections | 29 | - | **20.9** | **-** |
|  | Asymptomatic sub-microscopic infections | 19 | - | **13.7** | **-** |
|  | Total | 139 | - | **100.0** | **-** |
|  |  |  |  |  |  |
| **Annual *P. falciparum* parasitological index** | 278 *P. falciparum* cases per 1000 inhabitants |  |  |  |  |
|  |  |  |  |  |  |
