# Supplementary material for: Modelling the potential of focal screening and treatment as elimination strategy for Plasmodium falciparum malaria in the Peruvian Amazon Region
Source: Parasit Vectors. 2015 May 7;8:261. doi: 10.1186/s13071-015-0868-4 (PMC4429469; doi:10.1186/s13071-015-0868-4)
Supplement: Additional file 3: Text S1. — Description of transmission parameters for baseline model. [file 13071_2015_868_MOESM3_ESM.docx]

**Text S1. Description of transmission parameters for baseline model**

The susceptibility of an individual to infection (*b*) was assumed to be 0.05 based on estimates of two malariometric indices in Ninarumi: the annual *P. falciparum* force of infection in 2004 (*Pf-FOI* = 0.28 *P. falciparum* infections/person/year) and the annual *P. falciparum* entomological inoculation rate (*Pf-EIR* = 5.57 infective bites/person/year). The annual Pf-EIR was calculated by multiplying the annual-average *An. darlingi* human biting rate (Ad-HBR) by the *P. falciparum* sporozoite rate in *An. darlingi* mosquitoes (*Pf-SR*) and the number of days in a year (365). The annual Ad-HBR was estimated at 15.25 *An.darlingi* bites/person/night from mosquito collections undertaken in Ninarumi along 2003 [1]. Pf-SR was conservatively assumed to be 0.001 (0.1%), as observed in two entomological surveys carried out in 2008 in another area near Iquitos in the Peruvian Amazon [2].

The average human latent period (*l*) was set to 21 days based on previous reports about the biology of *P. falciparum* transmission stages [3]. The probability for an individual to develop symptoms upon infection and being detected by PCD was assumed to be 65% (*y*=0.65) based on results of the monitoring of symptoms in asymptomatic infections as part of the cohort study in Ninarumi [1]. ACT treatment effectiveness was assumed to be 98% (*ε*=0.98) according to an efficacy and effectiveness study of MQ-AS combination therapy for *P. falciparum* malaria in the Peruvian Amazon [4]. Daily recovery rate without treatment (*r_2_*) for *P. falciparum* was set to 1/200 based on case data compiled by Macdonald from several previous studies [5]. Daily recovery rate with ACT (*r_1_*) was conservatively assumed to be 1/14 according to antimalarial trials which measured gametocyte clearance from blood after treatment with MQ-AS [6-8] .

Susceptibility of *An. darlingi* was conservatively assumed to be 0.41 for *P. falciparum* based on published data for membrane-fed *An. darlingi* caught in Belize [9]. Considering an average annual temperature of 28°C for the Loreto region [10], the latent period for mosquito (*n*) (i.e. the sporogonic period) was estimated at 11 days for *P. falciparum* based on data from various experimental studies compiled and plotted against ambient temperature by Macdonald [11]. While daily human mortality rate (*h*) was set to 0.00005 considering an expected lifespan of 50 years at the median age of the population [12]; daily mosquito mortality rate (*g*) was assumed to be 0.16, after its estimation from the average parous rate [13] of *An. darlingi* in the Upper-Maroni forest region of French Guiana [14].

Basic malaria models have usually assumed arbitrary values between 1 and 100 for the mosquito density *(m)* (15), while the human feeding rate *(a)* is estimated by dividing the human blood index (proportion of mosquito-blood meals obtained from humans) by the duration of gonotrophic cycle in time units [16]. Due to large variations in values for both parameters, these were estimated by fitting the parameterized model to the observed monthly incidence of symptomatic *P. falciparum* infections in 2004 in Ninarumi. Setting a constant mosquito density *(m)* to 75, allowed the model to estimate monthly values of the human feeding rate *(a)* between 0.035 to 0.32 bites on humans per *An. darlingi* per day; a range considered realistic considering previously reported data on human blood indices (between 0.15 and 0.72 [17, 18]), and gonotrophic cycle periods (between 2.19 and 4.4 days [19, 20]) for *An. darlingi* in the Brazilian Amazon. Therefore, the human feeding rate *(a)* was the only time-dependent parameter in the model which allowed for the assessment of the seasonal variations of malaria transmission.

**References for parameters**

1. Branch O, Casapia WM, Gamboa DV, Hernandez JN, Alava FF, Roncal N, Alvarez E, Perez EJ, Gotuzzo E: **Clustered local transmission and asymptomatic *Plasmodium falciparum* and *Plasmodium vivax* malaria infections in a recently emerged, hypoendemic Peruvian Amazon community**. Malar J 2005, 4:27.
2. Parker BS, Paredes Olortegui M, Peñataro Yori P, Escobedo K, Florin D, Rengifo Pinedo S, Cardenas Greffa R, Capcha Vega L, Rodriguez Ferrucci H, Pan WK, Banda Chavez C, Vinetz JM, Kosek M: **Hyperendemic malaria transmission in areas of occupation-related travel in the Peruvian Amazon.** Malar J 2013, 12:178.
3. Day KP, Hayward RE, Dyer M: **The biology of *Plasmodium falciparum* transmission stages.** Parasitology 1998, 116 Suppl:S95-109.
4. de Oliveira AM, Chavez J, de Leon GP, Durand S, Arrospide N, Roberts J, Cabezas C, Marquiño W: **Efficacy and effectiveness of mefloquine and artesunate combination therapy for uncomplicated *Plasmodium falciparum* malaria in the Peruvian Amazon.** Am J Trop Med Hyg 2011;85(3):573-8.
5. Macdonald G, Goeckel GW: **The malaria parasite rate and interruption of transmission.** Bull World Health Organ 1964, 31:365-377.
6. Grande T, Bernasconi A, Erhart A, Gamboa D, Casapia M, Delgado C, Torres K, Fanello C, Llanos-Cuentas A, D'Alessandro U: **A randomised controlled trial to assess the efficacy of dihydroartemisinin-piperaquine for the treatment of uncomplicated falciparum malaria in Peru.** PLoS One 2007, 2(10):e1101.
7. Smithuis F, Kyaw MK, Phe O, Aye KZ, Htet L, Barends M, Lindegardh N, Singtoroj T, Ashley E, Lwin S, Stepniewska K, White NJ: **Efficacy and effectiveness of dihydroartemisinin-piperaquine versus artesunate-mefloquine in falciparum malaria: an open-label randomised comparison.** Lancet 2006, 367(9528):2075-85.
8. Sowunmi A, Nkogho OO, Okuboyejo TM, Gbotosho GO, Happi CT, Adewoye EO: **Effects of mefloquine and artesunate mefloquine on the emergence, clearance and sex ratio of *Plasmodium falciparum* gametocytes in malarious children.** Malar J 2009, 8:297.
9. Grieco JP, Achee NL, Roberts DR, Andre RG: **Comparative susceptibility of three species of Anopheles from Belize, Central America, to *Plasmodium falciparum* (NF-54).** J Am Mosq Control Assoc 2005, 21(3):279-90.
10. Aramburu Guarda J, Ramal Asayag C, Witzig R: **Malaria reemergence in the Peruvian Amazon region.** Emerg Infect Dis 1999, 5:209-215.
11. Macdonald G: **The analysis of the sporozoite rate.** Trop Dis Bull 1952, 49:569-586.
12. Instituto Nacional de Estadística e Informática: *Compendio Estadístico.* Lima: INEI; 2013.
13. Davidson G: **Estimation of the survival rate of anopheline mosquitoes in nature.** Nature 1954, 174:792-793.
14. Girod R, Gaborit P, Carinci R, Issaly J, Fouque F: ***Anopheles darlingi* bionomics and transmission of *Plasmodium falciparum*, *Plasmodium vivax* and *Plasmodium malariae* in Amerindian villages of the Upper-Maroni Amazonian forest, French Guiana.** Mem Inst Oswaldo Cruz 2008, 103:702-710.
15. McKenzie FE, Killeen GF, Beier JC, Bossert WH: **Seasonality, parasite diversity, and local extinctions in *Plasmodium falciparum* malaria.** Ecology 2001, 82(10):2673-2681.
16. Lardeux FJ, Tejerina RH, Quispe V, Chavez TK: **A physiological time analysis of the duration of the gonotrophic cycle of *Anopheles pseudopunctipennis* and its implications for malaria transmission in Bolivia.** Malar J 2008, 7:141.
17. Zimmerman RH, Galardo AK, Lounibos LP, Arruda M, Wirtz R: **Bloodmeal hosts of Anopheles species (Diptera: Culicidae) in a malaria-endemic area of the Brazilian Amazon.** J Med Entomol 2006, 43(5):947-56.
18. Charlwood JD: **Biological variation in *Anopheles darlingi* Root.** Mem Inst Oswaldo Cruz 1996, 91(4):391-8.
19. de Barros FSM, Honório NA, Arruda ME: **Survivorship of *Anopheles darlingi* (Diptera: Culicidae) in relation with malaria incidence in the Brazilian Amazon.** PLoS One 2011, 6:e22388.
20. Santos RL, Forattini OP, Burattini MN: **Laboratory and field observations on duration of gonotrophic cycle of *Anopheles albitarsis s.l*. (Diptera: Culicidae) in southeastern Brazil.** J Med Entomol 2002, 39(6):926-30.
