# Supplementary material for: Modelling the potential of focal screening and treatment as elimination strategy for Plasmodium falciparum malaria in the Peruvian Amazon Region
Source: Parasit Vectors. 2015 May 7;8:261. doi: 10.1186/s13071-015-0868-4 (PMC4429469; doi:10.1186/s13071-015-0868-4)
Supplement: Additional file 4: Text S2. — R-Code for the baseline model. [file 13071_2015_868_MOESM4_ESM.docx]

**Text S2. R-Code for the baseline model**

library(deSolve)

year<-20

tim<-360*year #time in days

time <- seq(0,tim,by=1)

es <- 1 # number of scenarios. It is only one scenario: the baseline model.

#Parameters

m <- 75 #mosquito density

Nt <-500 #total human population

Mt<- Nt*m #total mosquito population

b<- 0.05 #susceptibility of an individual to infection after being bitten by an infectious mosquito

c<- 0.41 #mosquito susceptibility to infection after biting an infectious individual

e<-0.98 #treatment effectiveness

y<-0.65 #fraction of symptomatic infections

r1<-14 #duration of infectious period in treated humans with ACT (in days)

r2<-200 #duration of infectious period in untreated humans in days (r2= r2a+r2b)

r2a<-80 #duration of presence of blood asexual parasites in untreated humans in days

r2b<-120 #duration of infectious period in untreated humans after clearance of blood asexual parasites in days

rr1<-1/r1; rr2<-1/r2; rr2a<-1/r2a; rr2b<-1/r2b

n<- 11 #duration of mosquito latent period in days

l<- 21 #duration of human latent period for *P.falciparum* in days (l= l1+l2+ne)

l1<-10 #duration from sporozoite inoculation to development of blood asexual parasites in days

l2<-10 #duration from asexual blood stage infection to development of gametocytes in days

rl2<-1/l2 #daily rate from appearance of blood asexual parasites to development of patent blood infection

ne<-1 #time spent in days (only one day) in a fictitious compartment called "New". “New” allows calculating daily incidence.

rnew<-1/ne #rate to enter/leave the fictitious compartment

h<- 1/(70*360) #daily human mortality rate

g<-0.16 #daily mosquito mortality rate

v<-1 #duration of FSAT in days

vv<-1/v

cov<-1 #coverage of intervention

sm1<-0.5 #sensitivity of microscopy for detecting asexual parasites

sp1<-0.95 #sensitivity of PCR for detecting asexual parasites

sm2<-0.1 #sensitivity of microscopy for gametocytes

sp2<-0.8 #sensitivity of PCR for detecting gametocytes

# monthly values of the human feeding rate (expected number of bites on humans per mosquito)

a<- rep(c(rep(0.32,30),rep(0.19,30),rep(0.04732,30),rep(0.12,30),rep(0.07,30),rep(0.095,30),rep(0.035,30),rep(0.035,30),rep(0.035,30), rep(0.035,30),rep(0.035,30),rep(0.035,30)),year)

a.t<-c(0,a)

A.t<-function(i) a.t[i]

# Scenario: Only passive case detection (PCD)

# Timing for the intervention

t1<-array(0,c(es,length(time)))

t1[1,]<-c(0,rep(0,360*year))

#Initial population number

To<-4

I1o<-9

I2o<-7

NEWo<-0

E1o<-0

E2o<-0

So<-Nt-I1o-I2o-E1o-E2o-To-NEWo

Vo<-0.001*Mt

Lo<-0

Uo<-Mt-Vo-Lo

#For storage

TIME<- array(0,c(es,length(time)))

Sx<- array(0,c(es,length(time)))

E1x<- array(0,c(es,length(time)))

NEWx<-array(0,c(es,length(time)))

E2x<- array(0,c(es,length(time)))

Tx<- array(0,c(es,length(time)))

I1x<- array(0,c(es,length(time)))

I2x<- array(0,c(es,length(time)))

Ux<- array(0,c(es,length(time)))

Lx<- array(0,c(es,length(time)))

Vx<- array(0,c(es,length(time)))

Nx<- array(0,c(es,length(time)))

Mx<- array(0,c(es,length(time)))

ITm<-array(0,c(es,((length(time)-1)/30)))

#Loop for scenarios

for(j in 1:es)

{

t1.t<-function(i) t1[j,][i]

#Function

mal1.dyn <- function(t,var,par) {

#Setting the lag for mosquito latent period

lagM <- t - n

if (lagM <= 0)

varlagM <- c(0,0,0,0,0,0,0,0,0,0) #"n" past values of compartments before time 0

else

varlagM <- lagvalue(lagM)

#Setting the lag for human latent period_1_

lagH <- t - l1

if (lagH <= 0)

varlagH <- c(0,0,0,0,0,0,0,0,0,0) #"l_1_" past values of compartments before time 0

else

varlagH <- lagvalue(lagH)

#Setting the time dependent gonotrophic cycle for a mosquito

a <- A.t(t+1)

#Setting the lag for gonotrophic cycle for a mosquito

if (lagM <= 0)

a_lagM <- 0

else

a_lagM <- A.t(t+1-n)

#Setting the lag for human latent period1

if (lagH <= 0)

a_lagH <- 0

else

a_lagH <- A.t(t+1-l1)

# Rename the variables and parameters

S<- var[1]

E1<- var[2]

NEW<- var[3]

E2<- var[4]

I1<- var[5]

I2<- var[6]

T<- var[7]

U<-var[8]

L<-var[9]

V<- var[10]

N<-S+E1+NEW+E2+NEW+I1+I2+T

M<-U+L+V

m <- par[1]

b <- par[2]

c<- par[3]

rr1<- par[4]

rr2a<- par[5]

rr2b<- par[6]

n<-par[7]

h<-par[8]

l1<-par[9]

l2<-par[10]

y<-par[11]

sm1<-par[12]

sp1<-par[13]

sm2<-par[14]

sp2<-par[15]

vv<-par[16]

g<-par[17]

e<-par[18]

cov<-par[19]

rnew<-par[20]

rl2<-par[21]

#Time dependent timing for the intervention

t1 <- t1.t(t+1)

# Rename Lags for mosquito and human latent period

S_lagM<-varlagM[1]

E1_lagM<-varlagM[2]

NEW_lagM<-varlagM[3]

E2_lagM<-varlagM[4]

I1_lagM<-varlagM[5]

I2_lagM<-varlagM[6]

T_lagM<-varlagM[7]

U_lagM<-varlagM[8]

L_lagM<-varlagM[9]

V_lagM<-varlagM[10]

S_lagH<-varlagH[1]

E1_lagH<-varlagH[2]

NEW_lagH<-varlagH[3]

E2_lagH<-varlagH[4]

I1_lagH<-varlagH[5]

I2_lagH<-varlagH[6]

T_lagH<-varlagH[7]

U_lagH<-varlagH[8]

L_lagH<-varlagH[9]

V_lagH<-varlagH[10]

# Derivatives

dS <- -m*a*b*(V/M)*S+rr1*T+rr2b*I2-h*S+h*(S+E2+E1+I1+I2+T)

dE1 <- m*a*b*(V/M)*S-m*(a_lagH)*b*(V_lagH/M)*S_lagH*exp(-l1*h)-h*E1

dNEW <- m*(a_lagH)*b*(V_lagH/M)*S_lagH*exp(-l1*h)-NEW*rnew

dE2 <- NEW*rnew-rl2*E2-h*E2-(sm1+sp1)*t1*cov*vv*e*E2

dI1 <- rl2*E2*(1-y*e)-rr2a*I1-h*I1-(sm1+sp1)*t1*cov*vv*e*I1

dI2 <- I1*rr2a-rr2b*I2-h*I2-(sm2+sp2)*t1*cov*vv*e*I2

dT <- rl2*E2*(y*e)-rr1*T-h*T+(sm1+sp1)*t1*cov*vv*e*E2+(sm1+sp1)*t1*cov*vv*e*I1+(sm2+sp2)*t1*cov*vv*e*I2

dU <- -a*c*((I1+I2+T)/N)*U+g*(V+L)

dL <- a*c*((I1+I2+T)/N)*U-(a_lagM)*c*((I1_lagM+I2_lagM+T_lagM)/N)*U_lagM*exp(-g*n)-g*L

dV <- (a_lagM)*c*((I1_lagM+I2_lagM+T_lagM)/N)*U_lagM*exp(-g*n)-g*V

list(c(dS,dE1,dNEW,dE2,dI1,dI2,dT,dU,dL,dV))} # Return the 10 values

#Preparing the numerical solution

par <- c(m,b,c,rr1,rr2a,rr2b,n,h,l1,l2,y,sm1,sp1,sm2,sp2,vv,g,e,cov,rnew,rl2)

init <- c(So,E1o,NEWo,E2o,I1o,I2o,To,Uo,Lo,Vo) #Initial population

time <- seq(0,tim,by=1)

#Numerical solution

mal1.sol <- dede(init,time,mal1.dyn,par)

#Relabeling the variables

TIME[j,]<- mal1.sol[,1]

Sx[j,]<- mal1.sol[,2]

E1x[j,] <- mal1.sol[,3]

NEWx[j,]<-mal1.sol[,4]

E2x[j,] <- mal1.sol[,5]

I1x[j,]<- mal1.sol[,6]

I2x[j,]<- mal1.sol[,7]

Tx[j,]<- mal1.sol[,8]

Ux[j,]<- mal1.sol[,9]

Lx[j,]<- mal1.sol[,10]

Vx[j,]<- mal1.sol[,11]

Nx[j,]<- Sx[j,]+E1x[j,]+E2x[j,]+NEWx[j,]+I1x[j,]+I2x[j,]+Tx[j,]

Mx[j,]<- Vx[j,]+Lx[j,]+Ux[j,]

#Monthly incidence calculation

for (x2 in 1:((length(time)-1)/30))

{

ITm[j,x2]<-sum(mal1.sol[2:(length(time)),4][(30*(x2-1)+1):(30*x2)])

}

}

#Plots

# 1. Prevalence of compartments

windows()

par(mfrow=c(2,1), xaxs='i', yaxs='i')

plot(Sx[1,]/Nt,type='l',xlab='time',main=paste('Prevalence'), ylab='Human population',col='blue',lwd=3,ylim=c(0,1))

lines((E1x[1,]+E1x[1,])/Nt, type='l',col='yellow', lwd=3)

lines((I1x[1,]+I2x[1,])/Nt, type='l',col='red', lwd=3)

lines(Tx[1,]/Nt, type='l',col='green', lwd=3)

legend("right", c("S","E","I","T"), col=c("blue","yellow","red","green"),lty=c(1,1,1,1),inset=0.01, cex=0.5)

plot(Ux[1,]/Mt,type='l', xlab='time',ylab='Vector population',col='green',lwd=3,ylim=c(0,1))

lines(Lx[1,]/Mt, type='l',col='purple', lwd=3)

lines(Vx[1,]/Mt, type='l',col='orange', lwd=3)

legend("right", c("U","L","V"), col=c("green","purple","orange"),lty=c(1,1,1), inset=0.01, cex=0.5)

###Figure 2...exported to TIFF file

##2. Prevalence and incidence by P. falciparum only 3 years

tiff(file = "PCDBaselineMAR1.tiff", width = 360*6, height = 360*6, units = "px", pointsize =6, res = 360)

par(mfrow=c(2,1))

par(mar=c(4,4.5,1,1))

# Figure 2A Incidence

observed<-rep(c(7,32,15,7,11,8,6,1,1,1,1,1),3) # Observed monthly incidence in Ninarumi

ax1<-seq(1,3*12,3)

ay1<-c("Y1 Jan","Apr","Jul","Oct","Y2 Jan","Apr","Jul","Oct","Y3 Jan","Apr","Jul","Oct")

leg<-c("Model-predicted overall incidence ","Model-predicted symptomatic incidence ",

"Observed symptomatic incidence")

INC<-ITm[1,(12*10+1):(12*(year-7))]

par(mar=c(4,4.5,1,1))

plot(INC*y, type="l",lty=1,lwd=2, col="blue", xaxt="n", xlab="", ylab="P.falciparum monthly incidence", ylim=c(0,max(INC*1.23)),cex=2)

text(1, max(INC*1.2),"A", cex=2, col="black", font=2)

lines(ITm[1,(12*10+1):(12*(year-7))],type="l", lty=1, lwd=2, col="dark gray")

lines(observed, type="l", lty=2, lwd=2, col="black")

legend(3.5,max(INC*1.24), leg, inset=0.004, cex = 1.1,lty = c(1,1,3), lwd=2, col=c("dark gray","blue","black"), horiz=F, y.intersp=1, box.col="white",ncol=2)

axis(1, at=ax1,labels=ay1, las=2, cex.axis=0.8, tck=-.02)

# Figure 2B Prevalence

ax1<-seq(1,3*360,13*7)

ay1<-c("Y1 Jan","Apr","Jul","Oct","Y2 Jan","Apr","Jul","Oct","Y3 Jan","Apr","Jul","Oct")

leg1<-c("Observed prevalence by microscopy","Model-predicted overall prevalence ","Model-predicted prevalence by microscopy")

leg2<-c("Model-predicted asymptomatic prevalence", "Model-predicted asymptomatic sub-microscopic prevalence",

"Model-predicted symptomatic prevalence")

cu<-(360*10+1):(360*(year-7))

prevPCD<-(E2x[1,cu]+I1x[1,cu]+I2x[1,cu]+Tx[1,cu])/Nt

eo<-c(rep(NA,360),rep(NA,60),6.5/100,rep(NA,29+180),1.1/100, rep(NA,89),rep(NA,360)) #Observed prevalence in Ninarumi

plot(prevPCD,type='l',xaxt="n", xlab="", lty=1, ylab='P.falciparum prevalence',col='dark gray',lwd=2,ylim=c(0,1.25*max(prevPCD)), cex=2)

text(0,1.23*max(prevPCD),"B", cex=2, col="black", font=2)

lines((E2x[1,cu]*0.5+I1x[1,cu]*0.5+I2x[1,cu]*0.1+Tx[1,cu])/Nt, type='l',col='dark gray', lty=2, lwd=2) #Microscopy prevalence

lines((E2x[1,cu]*0.35+I1x[1,cu]+I2x[1,cu])/Nt, type='l',col='red', lty=1, lwd=1.5) # Asymptomatic prevalence

lines((E2x[1,cu]*0.35*0.5+I1x[1,cu]*0.5+I2x[1,cu]*0.9)/Nt, type='l',col='red', lty=2, lwd=1.5) # Asymptomatic sub-microscopic prevalence

lines((E2x[1,cu]*0.65+Tx[1,cu])/Nt, type='l',col='blue', lty=1, lwd=1.5) #Symptomatic prevalence

#lines(NEWx[1,cu]/Nt, type='l',col='black', lwd=3.5)

points(1:(360*3),eo, pch ="*", bg = "black",cex=2)

legend(100,1.29*max(prevPCD), leg1, col=c("black","dark gray","dark gray"),lty=c(NA,1,3), pch=c("*",NA,NA),

inset=0.004, lwd=c(NA,2,2), cex=1.1, box.col="white",y.intersp=1, ncol=1)

legend(360*1.5,1.29*max(prevPCD), leg2, col=c("red","red","blue"),lty=c(1,3,1),

inset=0.004, lwd=c(2,2,2), cex=1.1, box.col="white",y.intersp=1, ncol=1)

axis(1, at=ax1,labels=ay1, las=2, cex.axis=0.8, tck=-.02)

text(c(460,675),c(0.065,0.01),c(0.065,0.01),cex = 1)

dev.off()
