# Supplementary material for: Modelling the potential of focal screening and treatment as elimination strategy for Plasmodium falciparum malaria in the Peruvian Amazon Region
Source: Parasit Vectors. 2015 May 7;8:261. doi: 10.1186/s13071-015-0868-4 (PMC4429469; doi:10.1186/s13071-015-0868-4)
Supplement: Additional file 5: Table S2. — One and five-year predicted cumulative number of P. falciparum infections averted after the addition of FSAT interventions to PCD at different timing, using either microscopy or PCR. [file 13071_2015_868_MOESM5_ESM.docx]

**Table S2. One and five-year predicted cumulative number of *P. falciparum* infections averted after the addition of FSAT interventions to PCD at different timing, using either microscopy or PCR**

|  |  |  |  | **Microscopy** | | | | |  | **Highly sensitivity test** | | | | |
| --- | --- | --- | --- | --- | --- | --- | --- | --- | --- | --- | --- | --- | --- | --- |
|  |  |  |  | **One year** | |  | **5 years** | |  | **One year** | |  | **5 years** | |
|  |  |  |  | **n** | **%** |  | **n** | **%** |  | **n** | **%** |  | **n** | **%** |
|  |  |  |  |  |  |  |  |  |  |  |  |  |  |  |
| **One FSAT** | |  |  |  |  |  |  |  |  |  |  |  |  |  |
|  | Month of implementation | January |  | 11 | 6.9 |  | 41 | 5.4 |  | 36 | 23.8 |  | 145 | 19.0 |
|  |  | February |  | 6 | 3.8 |  | 34 | 4.5 |  | 15 | 10.0 |  | 84 | 11.0 |
|  |  | March |  | 23 | 15.1 |  | 98 | 12.8 |  | 45 | 29.7 |  | 200 | 26.3 |
|  |  | April |  | 26 | 16.9 |  | 104 | 13.6 |  | 53 | 34.7 |  | 224 | 29.4 |
|  |  | May |  | 23 | 15.3 |  | 95 | 12.4 |  | 50 | 32.6 |  | 213 | 28.0 |
|  |  | June |  | 26 | 16.9 |  | 101 | 13.2 |  | 56 | 36.5 |  | 232 | 30.5 |
|  |  | July |  | 29 | 18.7 |  | 108 | 14.2 |  | 64 | 41.8 |  | 260 | 34.1 |
|  |  | August |  | 31 | 20.4 |  | 113 | 14.9 |  | 74 | 48.3 |  | 292 | 38.4 |
|  |  | September |  | 28 | 18.7 |  | 102 | 13.5 |  | 73 | 48.2 |  | 290 | 38.2 |
|  |  | October |  | 26 | 17.0 |  | 93 | 12.2 |  | 73 | 47.9 |  | 287 | 37.7 |
|  |  | November |  | 23 | 15.4 |  | 84 | 11.0 |  | 72 | 47.1 |  | 281 | 37.0 |
|  |  | December |  | 20 | 13.2 |  | 72 | 9.5 |  | 66 | 43.4 |  | 257 | 33.8 |
|  |  |  |  |  |  |  |  |  |  |  |  |  |  |  |
| **Two consecutive FSAT** | | |  |  |  |  |  |  |  |  |  |  |  |  |
|  | Time interval between FSAT | 7 days |  | 53 | 34.8 |  | 202 | 26.5 |  | 110 | 72.3 |  | 487 | 63.9 |
|  |  | 15 days |  | 53 | 34.5 |  | 201 | 26.3 |  | 110 | 72.6 |  | 489 | 64.3 |
|  |  | 30 days |  | 52 | 34.0 |  | 198 | 26.0 |  | 111 | 73.0 |  | 494 | 64.9 |
|  |  | 60 days |  | 50 | 33.0 |  | 192 | 25.2 |  | 111 | 73.0 |  | 497 | 65.3 |
|  |  | 90 days |  | 48 | 31.8 |  | 186 | 24.4 |  | 110 | 72.4 |  | 493 | 64.8 |
|  |  | 120 days |  | 46 | 30.2 |  | 176 | 23.1 |  | 107 | 70.3 |  | 477 | 62.6 |
|  |  | 150 days |  | 39 | 25.3 |  | 148 | 19.5 |  | 91 | 60.0 |  | 396 | 52.0 |
|  |  | 180 days |  | 35 | 22.7 |  | 145 | 19.1 |  | 80 | 52.4 |  | 356 | 46.7 |
|  |  |  |  |  |  |  |  |  |  |  |  |  |  |  |
| **Three consecutive FSAT** | | |  |  |  |  |  |  |  |  |  |  |  |  |
|  | Time interval between FSAT | 7 days |  | 68 | 45.0 |  | 271 | 35.7 |  | 127 | 83.7 |  | 598 | 78.5 |
|  |  | 15 days |  | 68 | 44.5 |  | 269 | 35.3 |  | 129 | 84.5 |  | 608 | 79.8 |
|  |  | 30 days |  | 66 | 43.5 |  | 263 | 34.6 |  | 130 | 85.1 |  | 617 | 81.1 |
|  |  | 60 days |  | 62 | 40.6 |  | 246 | 32.3 |  | 128 | 83.9 |  | 609 | 80.0 |
|  |  | 90 days |  | 51 | 33.8 |  | 217 | 28.5 |  | 113 | 74.5 |  | 535 | 70.3 |
|  |  |  |  |  |  |  |  |  |  |  |  |  |  |  |
